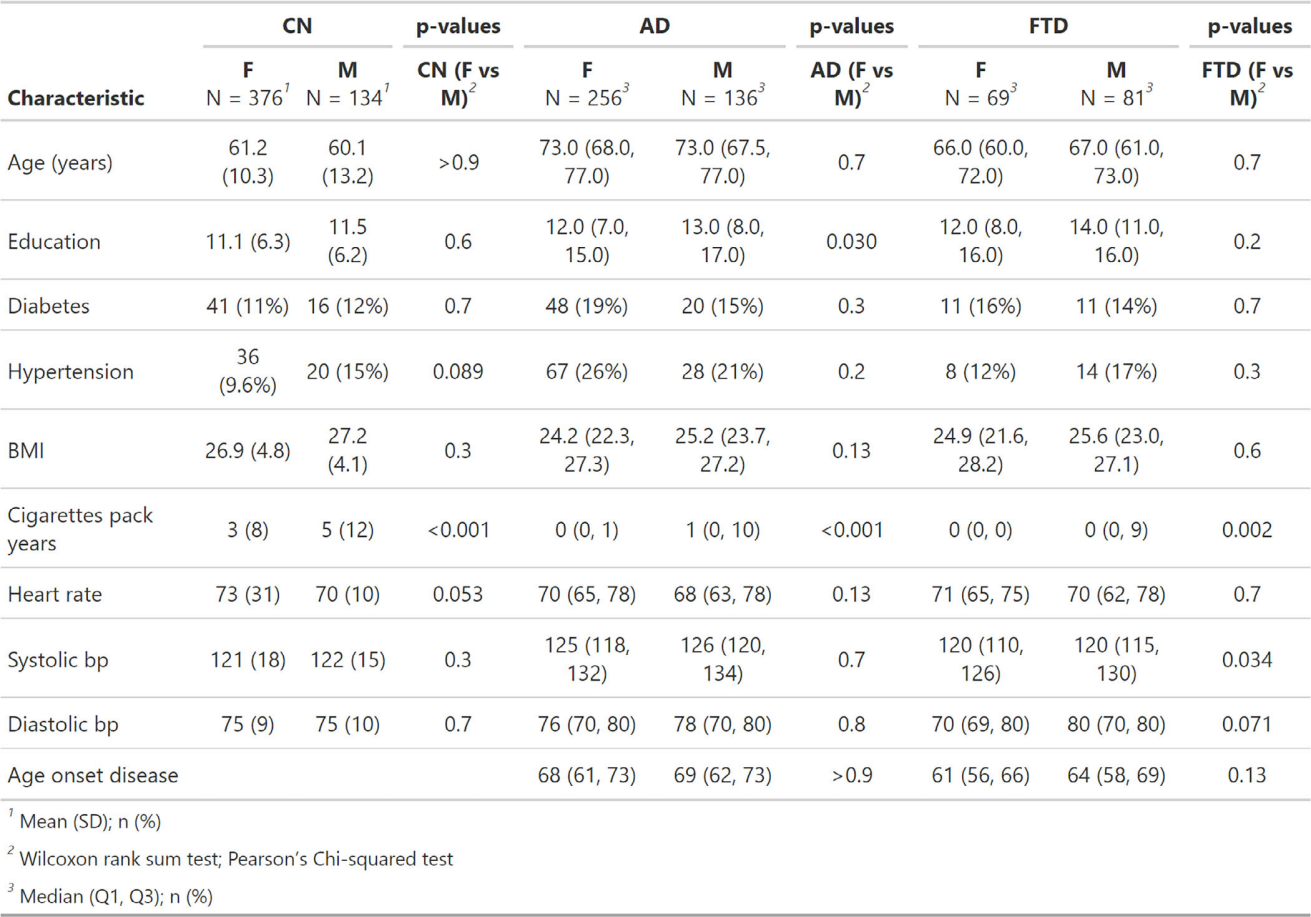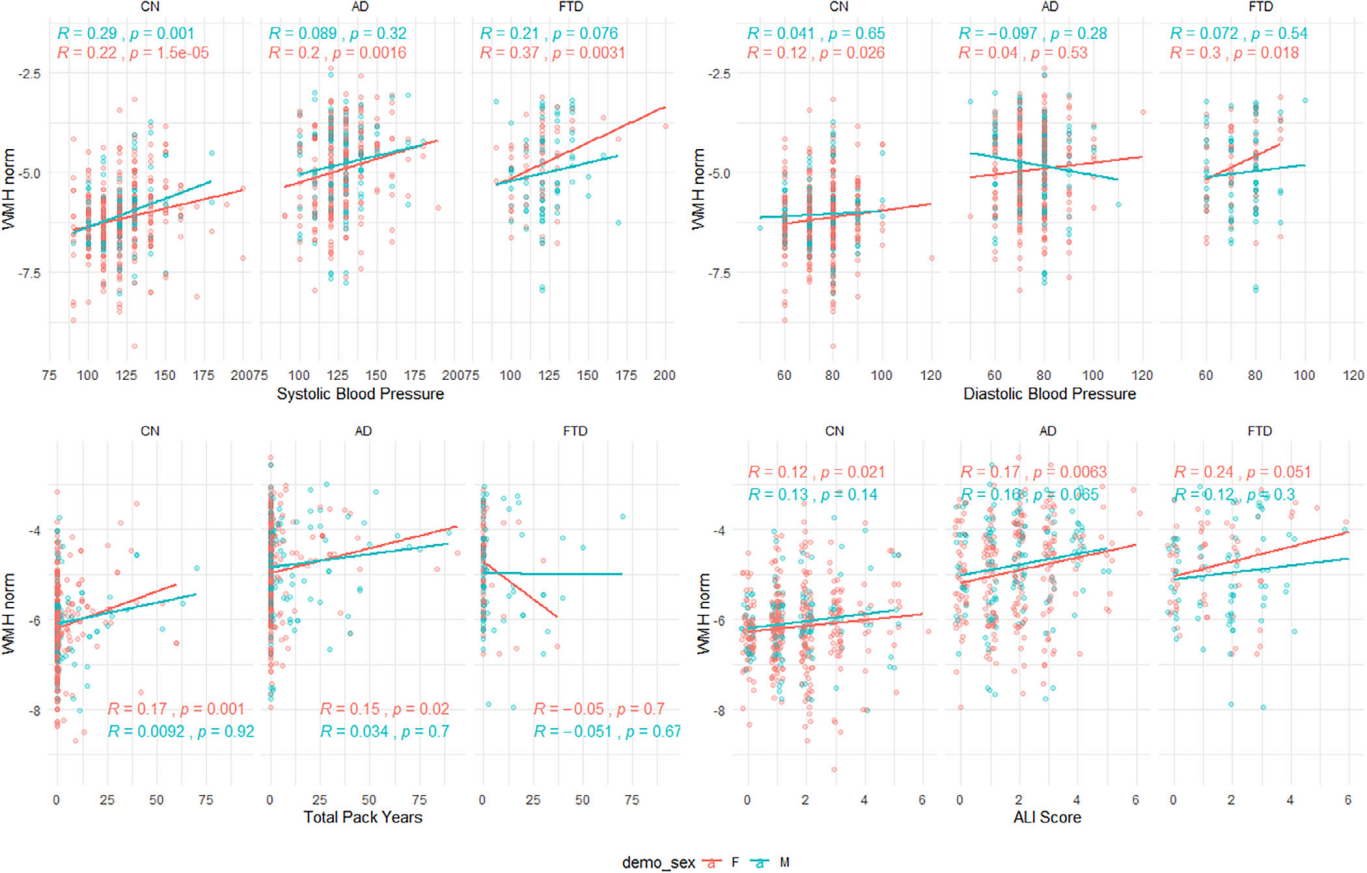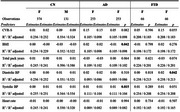# Sex Differences in the Association Between Cardiovascular Factors and White Matter Hyperintensities in a Latin American Cohort

**DOI:** 10.1002/alz70856_104855

**Published:** 2026-01-08

**Authors:** Florencia Altschuler, Veronica Canziani, Agustin Ibanez, Vicente Medel, Cecilia Gonzalez Campo

**Affiliations:** ^1^ Cognitive Neuroscience Center (CNC), Universidad de San Andrés, Buenos Aires, Buenos Aires, Argentina; ^2^ Universidad de Buenos Aires, Buenos Aires, Argentina; ^3^ Cognitive Neuroscience Center (CNC), Universidad de San Andres, Buenos Aires, Argentina; ^4^ Global Brain Health Institute (GBHI), Trinity College Dublin (TCD), Dublin, Dublin, Ireland; ^5^ Global Brain Health Institute (GBHI), University of California San Francisco (UCSF); & Trinity College Dublin, Dublin, Ireland; ^6^ Latin American Brain Health Institute (BrainLat), Universidad Adolfo Ibañez, Santiago, Chile; ^7^ Latin American Brain Health Institute (BrainLat), Universidad Adolfo Ibáñez, Santiago, Región Metropolitana de Santiago, Chile; ^8^ The University of Sydney, Sydney, NSW, Australia; ^9^ Universidad de Chile, Santiago, Chile; ^10^ CONICET, Buenos Aires, Argentina

## Abstract

**Background:**

Dementia incidence and progression differ between men and women, with women showing increased vulnerability. Sex differences in cerebrovascular pathology may contribute to variations in neurodegenerative disease progression. White matter hyperintensities (WMH), a marker of small vessel disease, are influenced by cardiovascular factors such as blood pressure (BP), heart rate (HR), body mass index (BMI), diabetes, and smoking. This study examines whether these factors have stronger associations with WMH in women compared to men across diagnostic groups: cognitively normal (CN), Alzheimer's disease (AD), and frontotemporal dementia (FTD).

**Method:**

We analyzed data from ReDLat, a consortium spanning across seven Latin American and Caribbean (LAC) countries, with 1,052 participants (66.7% women, mean age 66 years, SD = 10.5). The cohort included 510 CN, 392 AD, and 150 FTD patients. WMHs were quantified from T1 and FLAIR sequences using the Lesion Segmentation Toolbox (LST). WMH volumes were normalized and log‐transformed.

Cardiovascular risk was measured using a Cardiovascular Risk Score (CVR‐S) (0–6), where participants received one point if they were in the highest quartile for systolic or diastolic BP, HR, BMI, or smoking (total pack‐years), plus one point for a history of hypertension or diabetes. Associations between cardiovascular risk and WMH volume were analyzed, followed by sex‐stratified multiple regressions in CN, AD, and FTD groups, adjusting for age.

**Result:**

No significant sex differences were found in total WMH volume. However, systolic BP correlated with WMH in CN men and women, as well as in AD and FTD women. Diastolic BP correlated with WMH in CN and FTD women, while total pack‐years correlated with WMH in CN and AD women. CVR‐S was significantly associated with WMH burden in CN and AD women.

Regression models showed significant associations between systolic BP (AD women), diastolic BP (FTD women), HR (CN women), BMI (AD women), and total pack‐years (CN women) with WMH. A trend toward significance was observed between CVR‐S and WMH in FTD women.

**Conclusion:**

Cardiovascular risk factors show stronger associations with WMH in Latin American women, suggesting a sex‐specific vulnerability that may be influenced by social determinants of health. Addressing these disparities may help mitigate cerebrovascular contributions to neurodegeneration.